# Epidemiology of acute and chronic hepatitis B virus infection in Norway, 1992-2009

**DOI:** 10.1186/1471-2334-11-153

**Published:** 2011-05-26

**Authors:** Gražina Rimšelienė, Øivind Nilsen, Hilde Kløvstad, Hans Blystad, Preben Aavitsland

**Affiliations:** 1 European Programme for Intervention Epidemiology Training, European Centre for Disease Prevention and Control, Stockholm, Sweden; 2 Department of Infectious Disease Epidemiology, Norwegian Institute of Public Health, PO Box 4404 Nydalen, 0403 Oslo, Norway

## Abstract

**Background:**

Norway is classified as a low prevalence country for hepatitis B virus infection. Vaccination is only recommended for risk groups (intravenous drug users (IDUs), Men who have Sex with Men (MSM), immigrants and contacts of known carriers). We describe the epidemiology of reported cases of hepatitis B in Norway, during the years 1992-2009 in order to assess the validity of current risk groups and recommend preventive measures.

**Methods:**

We used case based data from the national surveillance system on acute and chronic hepatitis B. The Norwegian Statistics Bureau provided population and migration data and the Norwegian Institute for Alcohol and Drug Research the estimated number of active IDUs between 2002-2007. Incidence rates (IR) and incidence rate ratios (IRR) for acute hepatitis B and notification rates (NR) and notification rate ratios (NRR) for chronic hepatitis B with 95% confidence intervals were calculated.

**Results:**

The annual IR of acute hepatitis B ranged from 0.7/100,000 (1992) to 10.6/100,000 (1999). Transmission occurred mainly among IDUs (64%) or through sexual contact (24%). The risk of acquiring acute hepatitis B was highest in people aged 20-29 (IRR = 6.6 [3.3-13.3]), and in males (IRR = 2.4 [1.7-3.3]). We observed two peaks of newly reported chronic hepatitis B cases in 2003 and 2009 (NR = 17.6/100,000 and 17.4/100,000, respectively). Chronic hepatitis B was more likely to be diagnosed among immigrants than among Norwegians (NRR = 93 [71.9-120.6]), and among those 20-29 compared to those 50-59 (NRR = 5.2 [3.5-7.9]).

**Conclusions:**

IDUs remain the largest risk group for acute hepatitis B. The observed peaks of chronic hepatitis B are related to increased immigration from high endemic countries and screening and vaccination of these groups is important to prevent further spread of infection. Universal screening of pregnant women should be introduced. A universal vaccination strategy should be considered, given the high cost of reaching the target populations. We recommend evaluating the surveillance system for hepatitis B as well as the effectiveness of screening and vaccinating immigrant populations.

## Background

Two billion people have been exposed to the hepatitis B virus (HBV), 5 million cases of acute hepatitis B occur annually and over 350 million people have a chronic infection [1]. In total, hepatitis B results in 500,000-1.2 million deaths annually [1]. This carcinogenic virus causes 60-80% of the world's hepatocellular carcinoma. The risk is 25-35 times higher among those with chronic HBV infection [2] causing 300,000-500,000 deaths each year [1]. Ninety percent of infants infected during the first year of life and 30-50% of children infected between the ages of 1 and 4 develop chronic hepatitis B [3].

The annual incidence of reported hepatitis B in Europe varies from < 1 to 15/100,000 with the majority of the countries reporting < 5/100,000. Case definitions and classifications can play a role in this, as well as inclusion criteria of chronic cases into the data [4]. The prevalence of positive HBsAg tests in the general population of Europe varies by country from 0.1% to 7% [5].

Norway is generally a low prevalence country (0.5%) [6]. Certain recognised risk groups show a notably low prevalence, such as patients undergoing dialysis (< 1%) [7]. Low prevalence was also observed in pregnant women (0.1%) [8]. Due to the low prevalence, only a selective vaccination strategy is in place [9]. The alternative strategy, universal vaccination, will not have an effect on imported chronic hepatitis B cases, which have been perceived to represent the main disease burden. Vaccine is offered to well defined risk groups, such as injecting drug users (IDUs), contacts of known carriers, men who have sex with men (MSM), immigrants from countries with a high prevalence, medical workers and students, and newborns born to mothers from endemic countries with medium or high prevalence.

Despite the low reported incidence of hepatitis B in Norway, factors such as increasing intravenous drug use, as well as increased immigration and integration of immigrant communities are increasing the number of individuals at risk. We aimed to describe the epidemiology of reported cases of acute and chronic hepatitis B virus infection in Norway between 1992 and 2009 in order to assess the validity of current risk groups and recommend preventive measures.

## Methods

We described all cases reported to the Norwegian surveillance system for communicable diseases (MSIS) with a diagnosis of acute or chronic HBV infection between 1992 and 2009 by year of diagnosis and by sex, age, geographical location of infection, residence status, and county/municipality. Incidence rates (IR) and incidence rate ratios (IRR) with 95% confidence intervals (CI) for acute hepatitis B, as well as notification rates (NR) and notification rate ratios (NRR) of newly diagnosed cases of chronic infection were calculated using Excel, STATA v10.0 (STATA Corporation, College Station, TX, USA) and Episheet software.

### Existing surveillance system in Norway

Hepatitis B surveillance consists of two components: acute and chronic HBV infection. Acute hepatitis B has been a notifiable disease since 1975. In 1992, chronic HBV was also made notifiable. The reporting is based on mandatory notifications of laboratory confirmed cases from both clinicians and laboratories to the Norwegian Institute of Public Health (NIPH). The clinicians report the route of transmission, if known. At the NIPH, reports on the same patients are merged, data is validated and following the established case definition allocated, respectively to either the acute or chronic HBV databases. The case definition was modified in 2007 when HBV-RNA test result was added. The reporting and database is based on name and a unique personal identification number, so each report is validated against all previously registered information on the same person. In some cases, the NIPH contact the clinician to clarify information. Data collected includes mode of transmission, place of infection and clinical information. Sexual orientation is not specifically asked for on the notification form, but some clinicians report it. The national guidelines for screening of hepatitis B, recommend screening of immigrant populations from high endemic countries, particularly asylum seekers, including children adopted from abroad.

#### Case definitions

Cases are classified as acute hepatitis B in these two situations:

1: Cases with a laboratory-positive test result (positive HBsAg test result combined with a positive test result from at least one of the following microbiological tests: HBeAg, anti-HBc (IgG or IgM) antibody, HBsAg neutralisation test, or detection of HBV nucleic acid), and one of the following: 1) a clinically compatible case with an epidemiological link (known exposure to HBV) or 2) an acute hepatitis case without a known chronic hepatitis B virus infection or 3) another known reason for acute hepatitis.

2: Cases with anti-HBc seroconversion during the last 12 months combined with a positive test result in at least one of their most recent microbiological tests: HBsAg, HBV nucleic acid, or anti-HBs in the absence of vaccination against hepatitis B or treatment with specific immunoglobulin.

Cases are classified as chronic hepatitis B in this situation: cases who do not fulfil criteria for acute hepatitis B but who have a positive test result for HBsAg and anti-HBc antibody in the first available testing.

### Population

We used population and migration data from the Norwegian Statistics Bureau (SSB). The total population has increased from 4,273,634 in 1992 to 4,799,252 in 2009. For the analysis immigrants were considered as those born abroad and those born in Norway with at least one parent born abroad, asylum seekers and those with family reunification and other immigrants not specified above.

The estimated number of intravenous drug users (IDUs) (using the Mortality Multiplier method) was obtained from the Norwegian Institute for Alcohol and Drug Research (SIRUS), and available for the years 2002-2007.

Analysis by place of birth was performed for chronic hepatitis B in the period from 2003 to 2009 as this data is more accurate. Since 2003 place of birth is included in the MSIS database after linkage to the National Population Registry.

## Results

### Acute hepatitis B

**Figure 1:**
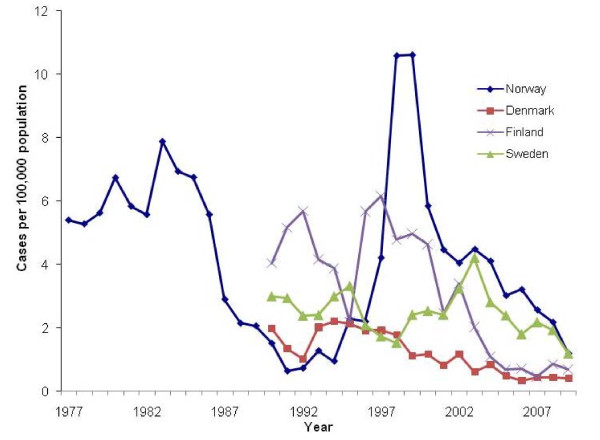
**Annual incidence of acute hepatitis B in Norway, 1992-2009**. Data from 1977 added for completeness of the figure; data for other Nordic countries included for comparison (http://data.euro.who.int/cisid; http://www.epinorth.org).

Before the period of our study and since the establishment of hepatitis B surveillance in Norway in 1975, a steady decrease in the incidence of acute hepatitis B was observed in early 1980's after the introduction of a selective vaccination strategy in 1984. During the study period in the late 1990s, a high incidence was again observed; this was due to an outbreak of acute hepatitis B and hepatitis A among IDUs [10]. Since 2000, there has been a decrease in the annual incidence from 5.9 to 1.2/100,000 in 2009 (Figure 1). Between 1992 and 2009 3,053 cases of acute hepatitis B were registered in the national surveillance system.

**Figure 2:**
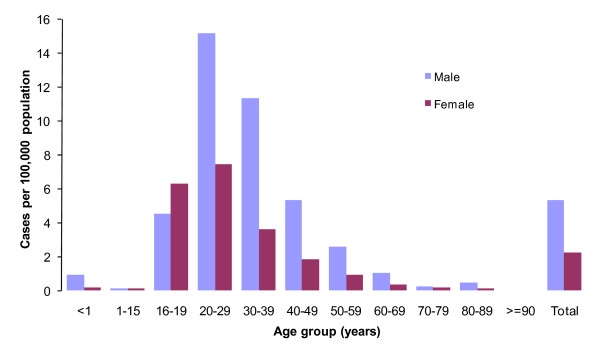
**Mean annual incidence of acute hepatitis B by age and sex, Norway, 1992-2009**. Blue bars represent number of males with acute hepatitis B per 100,000 population; purple bars represent females with acute hepatitis B per 100,000 population.

Acute hepatitis B was most common in men aged 20-40 years. Young people aged 20-29 were affected over 6 times more than those aged 50-59 (IRR = 6.6 [3.3-13.3]). Males were 2 times more likely to acquire the disease (IRR = 2.4 [1.7-3.3]) than females. People in the age group 16-19 years were 3 times more likely to acquire the disease as compared to those aged 50-59 years (Figure 2). Ninety percent of those who acquired acute hepatitis B were Norwegian and the annual incidence varied from 0.5 in 1992 up to 10.9/100,000 in 1999 and then back down to 1.2/100,000 population in 2009. As for immigrants, the incidence varied from 3.8/100,000 (1992) to 0.2/100,000 (2009) (IRR = 1.7 [0.8-3.5]). Most of the cases had acquired the disease in Norway (86%).

The main exposure for acute hepatitis B was the use of intravenous drugs (n = 1,963, 64%) followed by sexual activity (n = 718, 24%) of whom the majority (60%) did not specify sexual orientation, 29% reported heterosexual, and 11% homosexual activity. Exposure to infected blood (needle stick, transfusion/transplantation, nosocomial infection) accounted for less than 1% of cases (Table 1).

**Table 1:** Registered routes of transmission of acute and chronic hepatitis B, Norway 1992-2009

	Acute hepatitis B		Chronic hepatitis B	
**Route of transmission**	**Total reports**	**%**	**Total reports**	**%**
IDU	1963	64.3	415	4.2
Sexual activity:	718	23.5	408	4.2
heterosexual	207	28.8	8	0.1
homosexual	79	11.0	10	0.1
Sexual, unspecified	432	60.2	390	4.0
Mother-to-child	7	0.2	471	4.8
Nosocomial	7	0.2	20	0.2
Blood/transplantation	4	0.1	81	0.8
Needle stick	30	1.0	19	0.2
Other	21	0.7	174	1.8
Unknown	303	9.9	8212	83.8

Total:	3053	100	9800	100

Among those who reportedly acquired the disease through sexual activity 70% acquired it in Norway and 29% abroad. Among those infected abroad 48% were infected in Asia and 41% in European countries. Among those who reported being infected through heterosexual activity abroad 52% acquired it in Asia and 35% in Europe. For those reporting homosexual transmission the equivalent values were 18% and 70%, respectively. For unspecified sexual activity abroad 50% acquired it in Asia and 41% in Europe.

**Figure 3:**
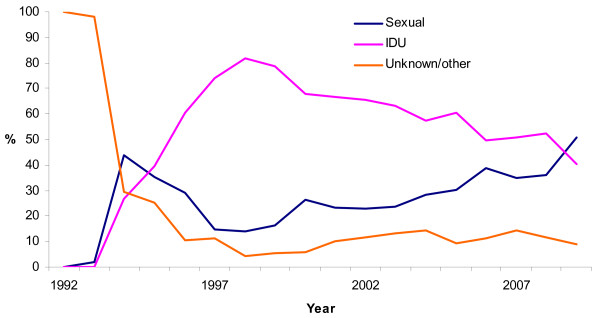
**Proportion of registered routes of transmission for acute hepatitis B, Norway, 1992-2009 (n = 3053)**. Proportion of routes of transmission for acute hepatitis B are presented with three colours: sexual route of transmission is presented with a dark blue line; a pink line represents injecting drug use and unknown and/or other routes of transmission are presented in orange colour.

The yearly number of cases of acute hepatitis B has decreased from 468 in 1998 to 57 in 2009. In 2009 intravenous drug use as the likely transmission route accounted for 40% of all acute hepatitis B cases and sexual transmission for 51%, during late 1990s this proportion was 82% and 14%, respectively (Figure 3). The pattern of acute hepatitis B among IDUs changed during the study period. The annual incidence ranged from 44/100,000 users in early 1990s to more than 3,500/100,000 users during an outbreak in 1999. This decreased to approximately 1000/100,000 users being infected per year in the period between 2000 and 2009. The majority were males (74%).

Acute infection is more often diagnosed in urban areas and more in the South. The highest proportion of intravenous drug users among acute cases was observed in Oslo (13%), Rogaland (11%) and Hordaland (10%) counties.

### Chronic hepatitis B

**Figure 4:**
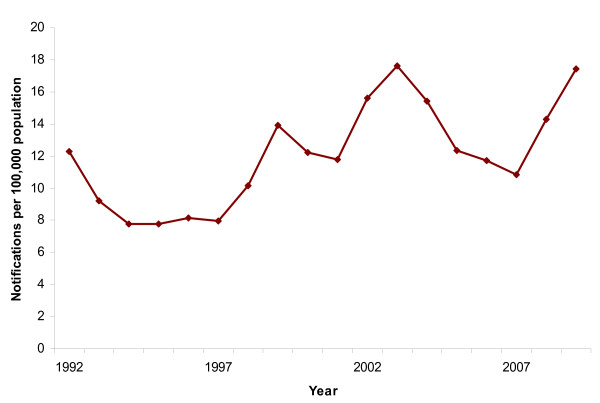
**Notification rate of chronic hepatitis B, Norway 1992-2009**.

From 1992 to 2009, 9,800 newly diagnosed cases of chronic hepatitis B have been registered. During the 1990s approximately 8/100,000 population of newly diagnosed chronic hepatitis B were registered annually. We observed three peaks in 1999, 2003 and 2009 (Figure 4).

Between 2003 and 2009, the reporting of country of birth for chronic infection improved from 16% (2003) to 85% (2009). Of those with known country of birth (n = 3,232), 78% were born in African (36%) and Asian (42%) countries. Of the African countries 58% were from Eastern Africa and 24% - Western Africa; from Asia there were 52% born in South-east Asia and 17% in Southern Asia. Altogether 12% of infected persons were born in Europe. Of those, 27% were born in Eastern Europe. 9% of all chronic hepatitis B cases were born in Norway (n = 284).

Immigrants were more than 90 times more likely to be newly diagnosed with chronic hepatitis B than Norwegians (NRR = 93 [71.9-120.6]). This was particularly evident for immigrants born abroad (NRR = 61 [46.7-80.4]). Chronic HBV infection among those born in Norway with at least one parent born abroad was less common (NR = 1/100,000 population; NRR = 0 [0-2.3]).

**Figure 5:**
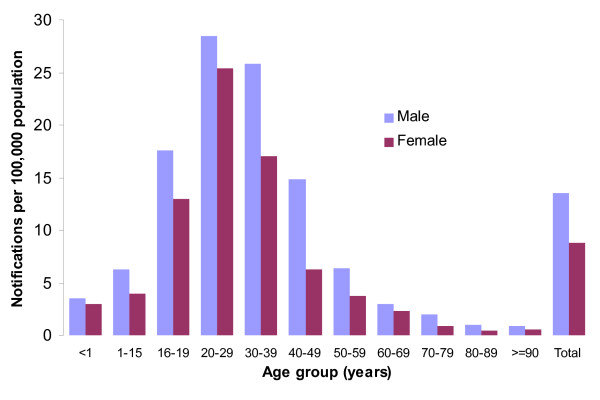
**Mean annual notification rate of chronic hepatitis B by age and sex, Norway, 1992-2009**. Blue bars represent number of notifications of chronic hepatitis B among males per 100,000 population; purple bars represent number of notifications of chronic hepatitis B among females per 100,000 population.

Chronic HBV infection was slightly more common among males than females (NRR = 1.5 [1.3-1.8]). It was also more common among those aged 20-40 years comparing to those aged 50-59 (20-29: NRR = 5.2 [3.5-7.9], 30-39: NRR = 4.2 [2.8-6.3]). An increase of the notification rate was observed in the age group 16-19 15.3/100,000 (Figure 5).

The exposure is not known in the majority (83%) of cases. Of the known factors, mother-to-child transmission was observed in almost 5% of the newly diagnosed cases, followed by injecting drug use (4%) and sexual activity (4%) (Table 1).

## Discussion

This study describes the epidemiology of acute hepatitis B and chronic HBV infection in Norway between 1992-2009. In accordance with the results from other European countries, acute hepatitis B most commonly occurs amongst injecting drug users [5,11]. Chronic infection is mostly affecting immigrants from high endemic parts of the world [6,12].

Nearly all the diagnosed cases of HBV infection are reported to the national surveillance system as laboratories mandatorily report all tests that have positive HBV markers. Clinicians also report a large proportion (80%) of the laboratory reported cases of acute hepatitis B. Therefore, there is a good overview on the disease in Norway. Nevertheless additionally to existing under-reporting only 30% of acute HBV cases would develop jaundice, which is the main reason to seek medical care among HBV patients [13].

There is no data in the surveillance system as to whether the person has been exposed to more than one risk factor (i.e. intravenous drug use and sex); although clinicians can report both. Nevertheless it is difficult to recognize which exposure caused the infection first. There is no data on HIV co-infection or other sexually transmitted infections.

Data for chronic HBV infection is not as accurate as for acute hepatitis B: exposure is unknown in 83% of notifications. It is difficult for clinicians to obtain information from immigrant populations from highly endemic countries, reflecting low reporting coverage.

The hepatitis B incidence in Norway is in the same range as other Nordic countries (Figure 1). Although, it is difficult to compare data from these countries as some surveillance systems do not distinguish between acute and chronic infection [4].

The increase in the late 1990s was due to a nationwide outbreak of hepatitis A and hepatitis B among IDUs [10,14]. Similar outbreaks were observed in Finland, Sweden and the Baltic countries, mainly among groups of non-immune IDUs [14,15]. These outbreaks coincided with increased number of IDUs and reduced drug prices [10,14,16].

While the estimated number of IDUs was stable between 2004 and 2008 [17], the number of IDUs with acute hepatitis B has decreased. According to the Norwegian Institute for Alcohol and Drug Research it might be a result of decrease in heroin users in the country which is probably related to a substantial increase in the availability of substitution treatment during the period [17]. Stabilization in the number of IDUs has also been observed in other European countries [18]. The decreasing incidence might also be due to vaccination of IDUs and their close contacts. Screening of a group of IDUs in Oslo (2009) indicates 35% vaccination coverage (unpublished data). The number of carriers (anti-HBc positive) among IDUs in Oslo has decreased from 50% in 2002 to 45% in 2007 [19]. In addition, following the large outbreak in late 1990s, the number of susceptible individuals has probably been considerably reduced.

Even though Norway is one of the countries with well established preventive measures for marginalised populations like IDUs, sexual activity with this risk group contributes to further spread of the infection. The proportion of cases infected through sexual activity has slightly increased in early 2000s following the outbreak among IDUs and has increased in 2009 (Figure 3). A larger proportion of males among those with acute hepatitis B is explained by the preponderance of males amongst IDUs. Surveillance data suggests sexual activity to be the second most important exposure. This is well recognized in other low prevalence countries [20,21]. For more than half of the cases of acute hepatitis B sexual orientation is not specified and this group is mostly represented by males (62%), partly covering all types of sexual orientation. The lack of information about sexual orientation is due to the ethical considerations of personal identification of the patient, therefore this data is not included in the notification form, preventing clinicians of asking for this kind of information.

Homosexual orientation was mentioned by 11% of those with known sexual orientation, all males. MSM are a well recognized risk group for hepatitis B and other STIs [11,22]. Vaccination is in place for this group in Norway as in other countries [4]. Although, vaccination coverage is not monitored, a web-based survey among MSM in Norway in 2007 [23] revealed a coverage of 47% among respondents (unpublished data). This is in line with a study from USA [24]. The injecting drug use among Norwegian MSM is unknown, however it is interesting to note that injecting drug use was uncommon among MSM recruited in the Amsterdam Cohort Studies [22].

Age distribution of the cases of acute hepatitis B with high incidence starting in age group 16-19 years reflects the onset of injecting drug use and sexual activity [17,25].

Although the mode of transmission was unknown in 83% of the chronic hepatitis B cases, we assume that almost all have been infected at birth or early in childhood [26].

The increase of newly diagnosed chronic hepatitis B in the last years of the study period is closely related to the increasing number of immigrants from countries with a higher prevalence of hepatitis B. The immigrant population represents 10% of the Norwegian population, with a twofold increase in residents of Asian, African, South and Central American origins since the early 90's, and a six fold increase in residents of Eastern European origin (SSB).

Immigrant populations from highly endemic countries have an impact on overall prevalence [12]. However, further infection from the immigrant population is quite limited [12]. Additionally, a molecular study by Fisker et al (2004) suggests that immigrants pose a smaller risk to general population than IDUs [20].

Hepatitis B screening of immigrants is in place in Norway. However, there is little data regarding the uptake of testing and vaccination amongst these populations. Certain immigrant populations are both hard to reach and to convince as to the merits of testing, this may be due to cultural differences.

Countries with selective vaccination strategy have low prevalence of the disease and behavioural risk groups are the main source of the infection. Low vaccination coverage among MSM and IDUs are in line with some studies, therefore suggesting a reconsideration of a universal vaccination strategy [21,27,28].

## Conclusions

We aimed to describe reported cases of acute and chronic hepatitis B virus infection in Norway between 1992 and 2009 in order to assess the validity of current risk groups and recommend preventive measures.

Norway currently has a low incidence of acute hepatitis B. Following an outbreak in the 1990s IDUs have remained the largest risk group for acute infection. Vaccination, availability of needles and syringes and the decreasing number of IDUs have probably contributed to the decreasing number of cases in this group.

Chronic HBV infection is closely related to the increase in the immigrant population from highly endemic countries. Therefore, screening of these populations and vaccination, if needed, is essential to prevent further spread of infection.

Screening of pregnant women, particularly immigrants from high and intermediate countries and contacts of IDUs, is necessary. Furthermore, given the high cost and logistical challenges of selective vaccination strategy, universal vaccination should be considered. Also, we recommend formal evaluation of the effectiveness of screening and vaccinating immigrant populations and of the hepatitis B surveillance system.

## Competing interests

The authors declare that they have no competing interests.

## Authors' contributions

GR performed data analysis and drafted the manuscript. ØN, HK, HB participated in the design of the study, helped to draft the manuscript. PA participated in design and coordination of the study. All authors read and approved the manuscript.

## Pre-publication history

The pre-publication history for this paper can be accessed here:

http://www.biomedcentral.com/1471-2334/11/153/prepub
